# A Phase Ib/II Study of Atezolizumab in Combination with Thiopurine Therapy in Patients with Metastatic Solid Tumors and Intermediate Tumor Mutational Burden

**DOI:** 10.1158/2767-9764.CRC-26-0210

**Published:** 2026-09-16

**Authors:** Christine Federspiel Secher, Martin Højgaard, Iben Spanggaard, Ane Yde Schmidt, Yuliu Guo, Luca Robinson, Daniela De Zio, Linea N. Toksvang, Frederik Otzen Bagger, Kjeld Schmiegelow, Kristoffer S. Rohrberg

**Affiliations:** 1 Department of Oncology, Copenhagen University Hospital, Rigshospitalet, Copenhagen, Denmark.; 2 MDxCore, Copenhagen University Hospital, Rigshospitalet, Copenhagen, Denmark.; 3 Cancer and Medicine, Danish Cancer Institute, Copenhagen, Denmark.; 4 Department of Cancer and Inflammation Research, Institute of Molecular Medicine, University of Southern Denmark, Odense, Denmark.; 5 Department of Pediatrics and Adolescents Medicine, Copenhagen University Hospital, Rigshospitalet, Copenhagen, Denmark.; 6 Department of Clinical Medicine, Faculty of Medicine, University of Copenhagen, Copenhagen, Denmark.

## Abstract

**Purpose:**

The Thiopurine Enhanced Mutations for PD-1/Ligand-1 Efficacy (TEMPLE) study was a phase Ib/II trial evaluating the safety, tolerability, and efficacy of atezolizumab combined with thiopurine therapy in patients with metastatic solid tumors, aiming to increase tumor mutational burden (TMB) through single-nucleotide mismatching and neoepitope generation.

**Patients and Methods:**

Phase Ib was an open-label, single-arm dose de-escalation trial conducted to determine the recommended phase II dose (RP2D) of 6-mercaptopurine (6MP) and 6-thioguanine (6TG) combined with atezolizumab in adult patients with metastatic solid tumors harboring intermediate TMB (5–10 mutations per megabase). Phase II used a Simon two-stage design to assess objective response per RECIST v1.1. Secondary endpoints included overall survival and progression-free survival.

**Results:**

In phase Ib, the initial dose level proved too toxic in this heavily pretreated population. The subsequent dose level (6MP 37.5 mg/m^2^ every day + 6TG 10 mg/m^2^ every day + atezolizumab 1,200 mg every 3 weeks) was well tolerated and defined as the RP2D. The most common treatment-related adverse events were anorexia, nausea, and fatigue. In phase II stage 1, 3 of 13 patients achieved stable disease, whereas the remaining 10 patients experienced progressive disease. No objective responses were observed, and the trial was terminated after stage 1. Translational analyses of serial tumor biopsies using whole-genome and RNA sequencing revealed no definitive shifts in TMB or neoepitope generation.

**Conclusions:**

A safe and tolerable dose of 6MP and 6TG combined with atezolizumab was identified in patients with metastatic solid tumors. Although clinical benefit was limited, the TEMPLE study informs future investigations in patients with less advanced disease.

**Significance:**

The TEMPLE study investigated a novel translational strategy aimed at increasing tumor immunogenicity through thiopurine-induced single-nucleotide mismatching, thereby enhancing neoepitope generation and potentially sensitizing tumors to PD-L1 blockade. A safe and tolerable combination treatment regimen was established; however, no objective responses were observed in this heavily pretreated and heterogeneous patient population.

## Introduction

Immune checkpoint inhibitors (ICI) targeting the PD-1/PD-L1 pathway have revolutionized cancer treatment by eliciting host immune responses and antitumor activity across multiple tumor types (1, 2). However, although some tumors respond remarkably well, most patients do not benefit from PD-1 blockade, emphasizing the need for novel therapeutic strategies.

Tumor mutational burden (TMB), defined as the number of nonsynonymous mutations per megabase (mut/Mb) of the tumor genome, is increasingly recognized as an emerging biomarker, with high TMB predicting a favorable response to ICIs (3, 4). High TMB (often defined as ≥10 mut/Mb) is associated with a higher number of neoantigens, thereby enhancing tumor immunogenicity (5). Although high TMB is frequently observed in malignant melanoma and non–small cell lung cancer, it remains uncommon in other major tumor types such as breast, colorectal, and prostate cancers (3).

The thiopurines 6-mercaptopurine (6MP) and 6-thioguanine (6TG) are purine antimetabolites widely used in the treatment of hematologic malignancies, including acute lymphoblastic leukemia (ALL; ref. 6). Their mechanism of action is the intracellular conversion of thiopurines into thioguanine nucleotides that are incorporated into the DNA as DNA-thioguanine (DNA-TG) in competition with guanine during DNA synthesis. Random S-methylation generating DNA methylguanine favors single-nucleotide mismatching, primarily DNA-TG·T mismatching (7). DNA-TG has been shown to be a strong predictor of relapse in observational studies of patients with ALL who were measurable residual disease positive after induction therapy (8). This finding led to the development of the Thiopurine Enhanced ALL Maintenance (TEAM) strategy, which increases DNA-TG by adding low-dose 6TG to a backbone of methotrexate and 6MP (9, 10).

Building on this mechanistic foundation, the Thiopurine Enhanced Mutations for PD-1/Ligand-1 Efficacy (TEMPLE) project investigates a novel translational approach designed to increase tumor immunogenicity through thiopurine-induced single-nucleotide mismatches, thereby promoting neoepitope generation and potentially enhancing sensitivity to PD-L1 blockade. The hypothesis is that thiopurine-based priming may be both tolerable and capable of improving ICI efficacy, thereby increasing the proportion of patients with otherwise ICI-resistant metastatic solid tumors who benefit from immunotherapy.

Here, we present results from the TEMPLE study, a phase Ib/II clinical trial evaluating the feasibility and potential clinical benefit of combining atezolizumab with 6MP and 6TG in adult patients with metastatic solid tumors exhibiting intermediate TMB (5–10 mut/Mb).

## Patients and Methods

### Study design

The TEMPLE study (NCT05276284) was a single-center, prospective phase Ib/II clinical trial designed to evaluate the safety, tolerability, and preliminary efficacy of atezolizumab in combination with the thiopurines 6MP and 6TG in patients with advanced and/or metastatic solid tumors having intermediate TMB, defined as 5 to 10 mut/Mb.

The phase Ib part of the study was an open-label, single-arm dose-escalation study aimed at determining the maximum tolerated dose (MTD) and/or the recommended phase II dose (RP2D) of 6MP and 6TG when combined with a fixed dose of atezolizumab. Dose de-escalation followed a modified 3 + 3 design. Patients were enrolled in cohorts of three, and dose decisions were based on the number of dose-limiting toxicities (DLT) observed during the 4-week DLT evaluation period. The highest dose level at which no more than one of six DLT-evaluable patients experienced a DLT was defined as the MTD and used as the RP2D after approval by the safety monitoring committee.

The phase II part was an open-label, single-arm study that followed a Simon two-stage design (11) to evaluate the antitumor activity of the combination therapy.

All patients provided written informed consent prior to enrollment. The study was conducted in accordance with the Declaration of Helsinki and approved by the Danish Ethics Committee (H-21076215; February 8, 2022) and the Danish Medicines Agency (EudraCT 2021-005327-20; January 26, 2022).

### Study population

Eligible patients were adults (≥18 years) with advanced and/or metastatic solid tumors, excluding primary central nervous system tumors, for whom no meaningful standard treatment options remained. Tumors were required to harbor an intermediate TMB based on local testing. Additional inclusion criteria included an Eastern Cooperative Oncology Group (ECOG) performance status of 0 or 1 and adequate organ function, as determined by standard laboratory assessments. Prior treatment with ICIs was permitted, provided that any immune-related adverse events (AE) had resolved to grade ≤1 before enrollment. Full inclusion and exclusion criteria are provided in Supplementary Table S1.

Exclusion criteria included deficiency in thiopurine methyltransferase (*TPMT*) or nudix hydrolase 15 (*NUDT15*)—genes known to influence the metabolism of thiopurines—which is why only patients with wild-type *TPMT* and *NUDT15* were included. In the initial protocol, patients who were heterozygous for *TPMT* were allowed; however, following the occurrence of a DLT, this criterion was revised in the first protocol amendment.

### Treatment, assessment, and evaluation

Due to the lack of known overlapping toxicities between thiopurines and atezolizumab, the initial starting dose was the full dose of both thiopurines and atezolizumab, with the option to de-escalate the dose if DLTs occurred. Potential dose levels were defined as follows:

1. Dose level 0 (DL0): 6MP 50 mg/m^2^ once daily + 6TG 12.5 mg/m^2^ once daily + atezolizumab 1,200 mg every 3 weeks.
2. Dose level −1 (DL−1): 6MP 37.5 mg/m^2^ once daily + 6TG 10 mg/m^2^ once daily + atezolizumab 1,200 mg every 3 weeks.
3. Dose level −2 (DL−2): 6MP 25 mg/m^2^ once daily + 6TG 7.5 mg/m^2^ once daily + atezolizumab 1,200 mg every 3 weeks.

Treatment with 6MP and 6TG was initiated 1 week prior to the first dose of atezolizumab. The combination regimen was administered in 21-day cycles. No dose escalation was planned. Treatment could be continued for up to 2 years until disease progression, unacceptable toxicity, or patient withdrawal.

In phase Ib, the primary objectives were to assess safety and tolerability and to determine the MTD and/or the RP2D of 6TG and 6MP in combination with fixed-dose atezolizumab.

In phase II, the primary endpoint was antitumor activity, measured as the objective response rate according to RECIST 1.1. Treatment response was evaluated across all known disease sites using appropriate imaging modalities, primarily CT, every 9 weeks. Patients were considered evaluable for endpoint analyses if they had completed more than one cycle of treatment and had received more than 80% of the planned thiopurine doses. Secondary endpoints included progression-free survival (PFS), overall survival (OS), and duration of response.

All AEs were monitored continuously and graded according to the Common Terminology Criteria for Adverse Events (CTCAE), version 5.0.

### Statistical analyses

Phase Ib planned to include 3 to 18 patients, depending on the number of DLTs observed and the need for dose de-escalation. Patients treated at the RP2D during phase Ib were included in the primary endpoint analyses of phase II and contributed to the decision to proceed to stage 2.

Phase II followed a Simon minimax two-stage design (11), with 13 patients in stage 1 and 14 patients in stage 2. One objective response among the first 13 patients was required to proceed to stage 2 of phase II.

PFS and OS were analyzed using the Kaplan–Meier method, with medians and 95% confidence intervals (CI) reported. Statistical analyses were conducted in R (version 4.4.2).

No statistical tests were performed on the translational on-treatment biopsies due to the limited sample size.

### Pharmacodynamic markers of thiopurine treatment

Levels of DNA-TG in crude DNA from circulating leukocytes, thioguanine nucleotide concentrations in erythrocytes, and methylated 6MP metabolites in erythrocytes were measured weekly during the first 2 months of treatment, according to the procedures described by Jacobsen and colleagues (12).

### Biopsies and genomic data

Fresh tumor biopsies were obtained at baseline, after 28 days of treatment, and after 3 months. Three samples were collected from the same lesion. Two were preserved in RNAlater for genomic analyses, and one was formalin-fixed and paraffin-embedded for histopathologic evaluation. At baseline, tumor biopsies and matched normal blood samples were collected from each patient to enable subtraction of germline variants. Tumor samples were sequenced using both whole-genome sequencing (WGS) and RNA sequencing (RNA-seq), whereas normal samples were sequenced using WGS only.

For WGS, a standard input of 400 ng DNA (minimum 100 ng) purified from either whole blood or tumor tissue was used as the input for the Illumina DNA PCR-Free Prep protocol. Sequencing was performed on either the Illumina NovaSeq 6000 or NovaSeq X Plus, obtaining a minimum coverage of 30× for germline samples and 60× (aiming for 90×) for tumor samples, with at least 95% of the genome covered ≥10×. Somatic variants were called using Strelka2 v2.7 in a tumor–normal paired setting (13) and annotated using Ensembl Variant Effect Predictor (VEP) v104.3 (14). We retained nonsynonymous variants, including in-frame insertions and deletions, frameshift mutations, and missense mutations. Based on the VEP annotations, protein-level changes were inferred for each variant.

For tumor RNA-seq, RNA was processed using the Illumina Stranded Total RNA Prep kit, with 50 to 100 ng as the standard input. RNA libraries were sequenced on an Illumina NovaSeq 6000 platform.

Patients were included based on an intermediate TMB value determined by local testing. TMB was subsequently reevaluated by central review using WGS.

To assess the pharmacodynamic effect of thiopurine treatment, changes in similarity to the COSMIC thiopurine chemotherapy–associated mutational signature SBS87 were evaluated (15).

### T cell–inflamed gene expression profile score calculation

The T cell–inflamed gene expression profile (GEP) score is a pan-cancer RNA signature comprising 18 genes indicative of a T cell–activated tumor microenvironment. A higher GEP score indicates a more inflamed tumor microenvironment and has been associated with an increased likelihood of response to PD-1 blockade across multiple tumor types (16). This score was used to assess immune infiltration. To quantify the T cell–inflamed GEP, we followed a previously described method (16, 17). RNA-seq reads were aligned to the GRCh38 reference genome using STAR v2.7.11b (18), and gene-level raw count matrices were imported into R (version 4.2.0) for downstream analysis.

### Neoepitope selection

To capture neoepitopes arising from somatic single-nucleotide variants (SNV) and gene fusions, patient-specific mutated proteomes were constructed by applying each patient’s variant calls and the corresponding predicted amino acid changes to the reference proteome.

For each mutated protein sequence, candidate 9-mer peptides encompassing the mutated residue were generated using a sliding window approach and exported in FASTA format. Aligned RNA-seq BAM files were used to quantify variant allele frequencies (VAF) at loci identified by WGS, and only peptides derived from variants supported by more than one RNA-seq read were retained. Gene fusions were called from RNA-seq data using Arriba v2.4.0 (19) and filtered to those with expression supported based on STAR-Fusion v1.14.0 (https://www.biorxiv.org/content/10.1101/120295v1). HLA typing was performed with T1K v1.0.5 (20). All mutated peptides were evaluated using NetMHCpan v4.1 (21) with default settings, and peptides with an eluted ligand percentile rank <1% were retained as predicted MHC class I neoepitopes.

## Results

**Figure 1.**
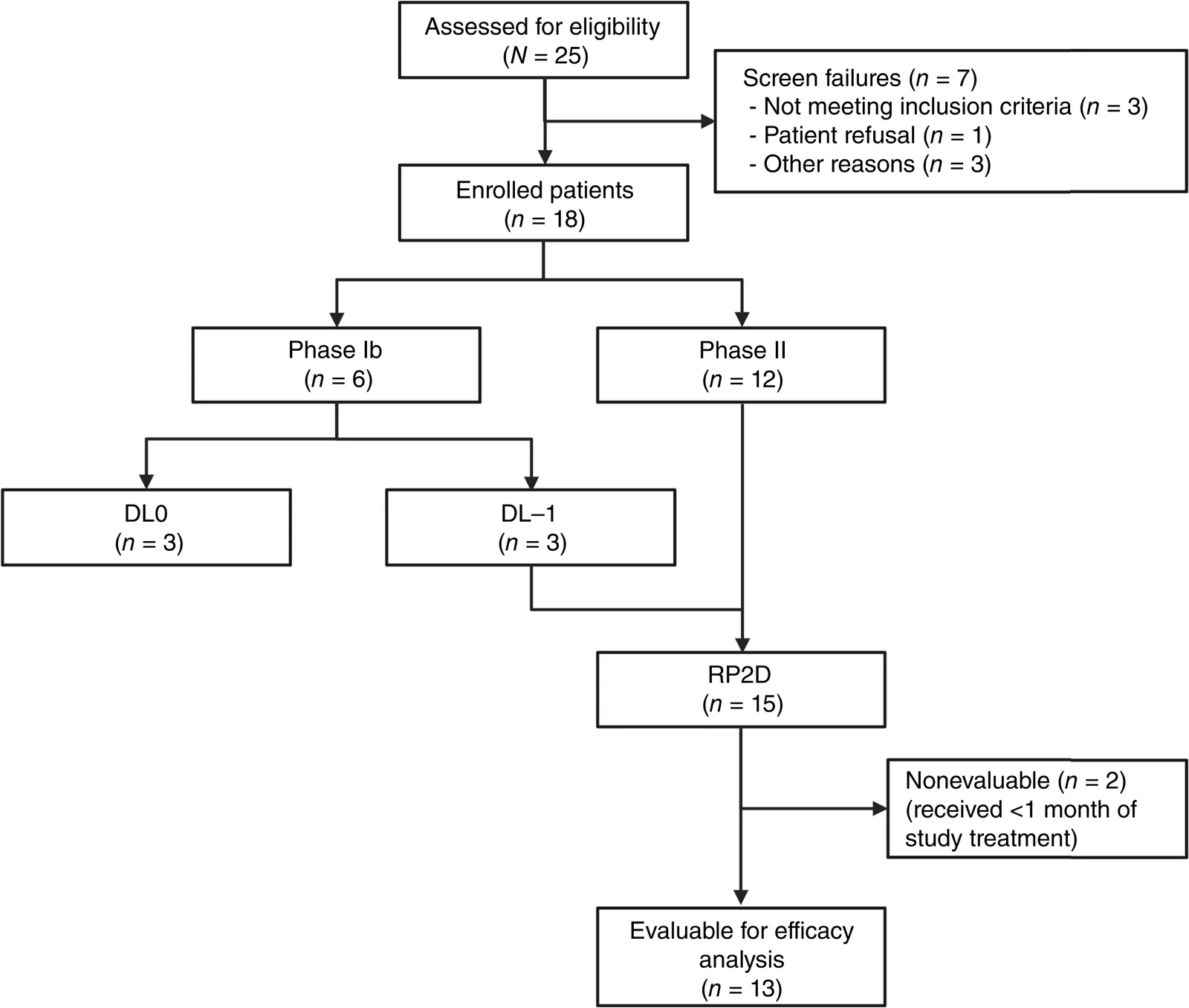
CONSORT diagram.

A total of 18 patients were enrolled in the TEMPLE study between September 2022 (first patient first visit) and December 2024 (last patient last visit; Fig. 1).

All patients had advanced-stage metastatic solid tumors. The median age was 62.5 years (range, 33–78), and 10 patients (55.6%) were female. Baseline characteristics of all treated patients are summarized in Table 1. Most patients had colorectal cancer (*n* = 7) or breast cancer (*n* = 4). Other tumor types included esophageal cancer (*n* = 2), small cell lung cancer (*n* = 2), dendritic cell sarcoma (*n* = 1), large cell neuroendocrine carcinoma of the lung (*n* = 1), and ovarian cancer (*n* = 1).

**Table 1.** Baseline characteristics.

Characteristic	Overall (*N* = 18)
Age, median (range)	62.5 (33–78)
Gender, *n* (%)	​
Female	10 (55.6)
Male	8 (44.4)
Tumor type, *n* (%)	​
Colorectal cancer	7 (38.9)
Breast cancer	4 (22.2)
Esophageal cancer	2 (11.1)
Small cell lung cancer	2 (11.1)
Dendritic cell sarcoma	1 (5.6)
Large cell neuroendocrine carcinoma of the lung	1 (5.6)
Ovarian cancer	1 (5.6)
TMB at entry (local testing), median (range)	7.3 (5.2–9.5)
TMB at entry (central review), median (range)	5.6 (2.2–10.6)
ECOG performance status at baseline, *n* (%)	​
0	12 (66.7)
1	6 (33.3)
Number of prior treatment lines, median (range)	4 (2–13)
Prior treatment with immunotherapy, *n* (%)^a^	4 (22.2)

a Prior immunotherapy treatment included investigational anti-TNFR2 antibody, atezolizumab, and pembrolizumab.

The median number of prior systemic treatment lines was 4 (range, 2–13), and four patients (22.2%) had previously received immunotherapy, including an investigational anti-TNFR2 antibody, the PD-1 inhibitor pembrolizumab, and the current study drug, the PD-L1 inhibitor atezolizumab. At baseline, the ECOG performance status was 0 in 12 patients (66.7%) and 1 in 6 patients (33.3%). TMB at study entry was a median of 7.3 mut/Mb (range, 5.2–9.5) based on local testing and 5.6 mut/Mb (range, 2.2–10.6) based on subsequent central review.

### Phase Ib

Six patients were enrolled in phase Ib. Three patients received treatment at DL0. At this dose level, all three patients developed neutropenia (two patients with CTCAE grade 1 and one patient with grade 4), and one patient was admitted with febrile neutropenia and sepsis. Several other AEs, including nausea, fatigue, and increased liver tests, were also observed. In addition, DNA-TG levels in blood increased to high concentrations (1,250–2,500 fmol/μg DNA) within the first 2 weeks of treatment (Supplementary Fig. S1A) although no clear association with AEs, including nausea and neutropenia, could be shown. Due to the toxicity profile and the high concentrations of DNA-TG, the dose-escalation committee decided to de-escalate to DL−1.

The subsequent three patients were treated at DL−1. At this dose level, no DLTs were observed. Neutrophil counts remained within the reference range, and DNA-TG levels consistently exceeded the predefined target of 500 fmol/μg DNA (Supplementary Fig. S1B). Two grade 3 liver function test abnormalities were observed, consistent with the known toxicity profile of 6MP treatment. The overall toxicity profile at DL−1 was considered acceptable. Based on these findings, DL−1 was established as safe and tolerable and was selected as the RP2D.

### Phase II

In phase II, the three patients treated at the RP2D during phase I were included in the final analysis. Of the 15 patients treated at the RP2D, 13 patients were evaluable for the primary endpoint analyses, having completed more than one cycle of treatment.

**Figure 2.**
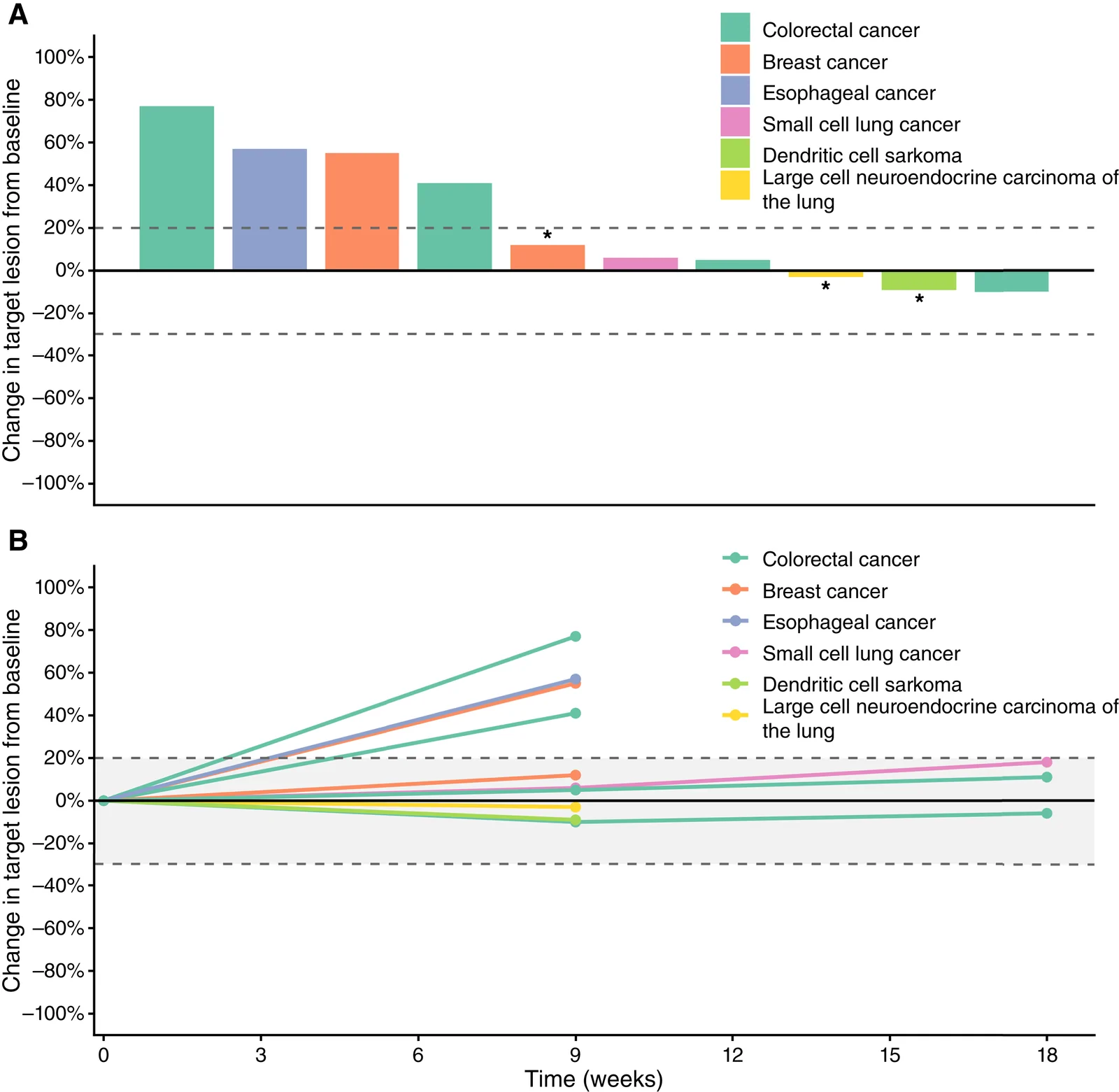
**A,** Maximum percentage change from baseline in target lesion size. The asterisk indicates the simultaneous appearance of new lesions. **B,** Spider plot of percentage change from baseline in tumor burden during treatment.

Three patients (23%) achieved stable disease at the first tumor assessment, whereas the remaining 10 patients (77%) were defined as having progressive disease. As no objective responses were observed, the trial was terminated after the completion of stage 1, in accordance with a Simon two-stage design. Best responses and treatment duration for evaluable patients with available assessment scans (*n* = 10) are illustrated in Fig. 2.

Of note, although classified as having progressive disease according to RECIST criteria, two patients demonstrated evidence of antitumor activity on imaging scans, showing a mixed response with tumor shrinkage at some sites (lung metastases and pericardial lymph node) and disease progression at others.

**Figure 3.**
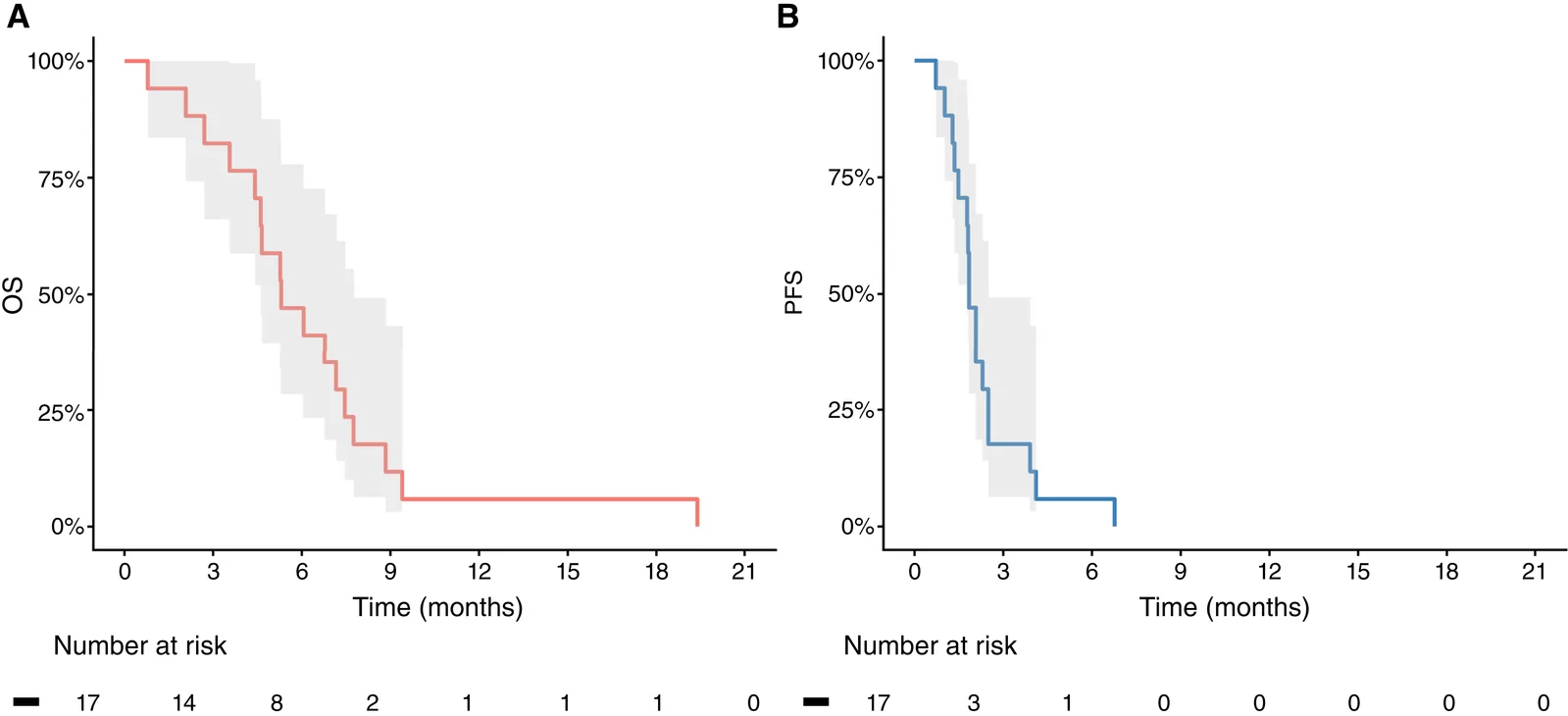
Kaplan–Meier curves for (**A**) OS and (**B**) PFS, with 95% CIs.

Secondary endpoints, including OS and PFS, are presented in Fig. 3A and B for all patients who received study treatment (*n* = 17). The median OS was 5.3 months (95% CI, 4.6–7.8), and the median PFS was 1.8 months (95% CI, 1.8–2.5). One of the nonevaluable patients was started on thiopurine treatment [cycle 1 day −7 (C1D–7)] but never initiated atezolizumab (C1D1) and was therefore not included in the survival analyses.

### Safety

In total, 118 AEs were reported during the study, including nine serious AEs (SAE). A summary of all AEs is provided in Supplementary Table S2.

In phase I at DL0, a total of 22 AEs were reported, the majority of which (*n* = 19, 86.4%) were grade 1 to 2, whereas three AEs (13.6%) were grade ≥3. In phase I, two SAEs occurred (febrile neutropenia and sepsis), both of which were deemed related to treatment with 6MP and 6TG.

At DL−1/RP2D, a total of 96 AEs were reported, the majority (*n* = 88, 91.7%) being grade 1 to 2, whereas eight AEs (8.3%) were grade ≥3. Seven SAEs occurred, of which only one was considered related to 6MP and 6TG: In a patient with metastatic breast cancer and extensive liver involvement, a case of suspected sinusoidal obstruction syndrome (SOS) occurred, a well-known complication of treatment with thiopurines.

No SAEs were considered related to atezolizumab at either dose level. Six AEs (5.1%) were considered related to atezolizumab during the entire study; these included rash, pruritus, increased fatigue, flushing, fever, and infusion-related reaction. All six related events were categorized as grade 1 or 2 (Supplementary Table S2). No unexpected immune-related AEs were observed during the study.

The three most common treatment-related AEs were anorexia, nausea, and fatigue, consistent with the expected safety profile. All treatment-related AEs are presented in Table 2, and an overview of the most common AEs observed during the study (>10% in total patients) is shown in Supplementary Table S3. All cases of neutropenia occurred at DL0, with none observed after de-escalation to DL−1. Elevated liver enzymes, a well-known adverse effect of thiopurines, were observed in several patients; however, only one case of grade 3 alanine aminotransferase elevation (in the patient with suspected SOS) and three cases of grade 3 aspartate aminotransferase elevation were reported.

**Table 2.** Treatment-related AEs in all included patients.

Term	All grades	Grade ≥ 3
Anorexia, *n* (%)	6 (33%)	​
Nausea, *n* (%)	5 (28%)	​
Fatigue, *n* (%)	3 (17%)	​
Dysgeusia, *n* (%)	2 (11%)	​
Febrile neutropenia, *n* (%)	1 (6%)	1 (6%)
Drug induced liver injury, *n* (%)	1 (6%)	1 (6%)
Dry mouth, *n* (%)	1 (6%)	​
Fever, *n* (%)	1 (6%)	​
Flushing, *n* (%)	1 (6%)	​
Infusion-related reaction, *n* (%)	1 (6%)	​
Pruritus, *n* (%)	1 (6%)	​
Rash, *n* (%)	1 (6%)	​
Ructus, *n* (%)	1 (6%)	​
Sepsis, *n* (%)	1 (6%)	1 (6%)
Stomatitis, *n* (%)	1 (6%)	​
Biochemistry:	​	​
Anemia, *n* (%)	8 (44%)	​
Liver function tests^a^, *n* (%)	4 (22%)	3 (17%)
Neutrophil count decreased, *n* (%)	3 (17%)^b^	1 (6%)^b^
Thrombocytopenia, *n* (%)	1 (6%)	1 (6%)

a Liver function tests cover all reported CTCAE-defined liver toxicities, including transaminase increases, SOS, and gamma-glutamyl transferase elevation.

b All incidents of neutropenia were observed in the phase I patients receiving the high starting dose DL0.

### Pharmacodynamic markers of thiopurine treatment

Levels of DNA-TG during the study are shown in Supplementary Fig. S1A and S1B.

Supplementary Figure S1A shows DNA-TG levels in the three patients treated in phase Ib, initially at DL0 and subsequently at DL−1. At DL0, very high DNA-TG levels (>1,200 fmol/μg DNA) were observed, classified as high when compared with the DNA-TG levels observed in patients with ALL treated with 6MP and 6TG (9). Following treatment pause and dose reduction, DNA-TG levels decreased to lower ranges.

Supplementary Figure S1B shows 15 patients treated in phase II at the RP2D (DL−1), demonstrating that DNA-TG levels consistently exceeded the predefined target of 500 fmol/μg DNA.

### Translational analyses

**Figure 4.**
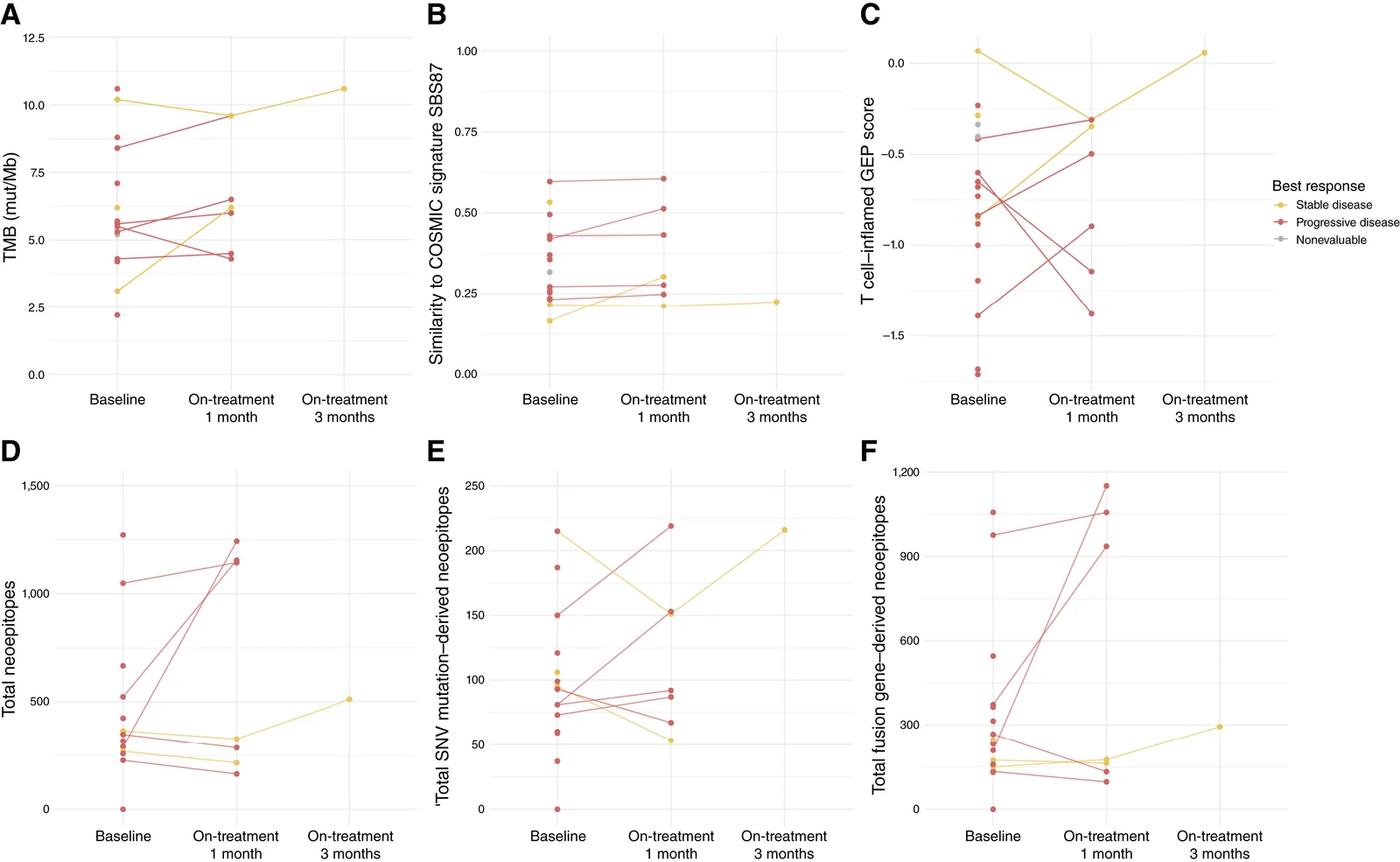
Translational analyses of baseline and on-treatment biopsies collected after 1 and 3 months of treatment. **A,** TMB. **B,** COSMIC thiopurine chemotherapy–associated mutational signature SBS87. **C,** T cell–inflamed GEP score. **D,** Total neoepitope count. **E,** Mutation-derived neoepitopes. **F,** Fusion gene–derived neoepitopes.

In total, 18 baseline and seven on-treatment biopsies were analyzed and are visualized in Fig. 4 according to best treatment response. Figure 4A shows the changes in TMB, and Fig. 4B shows the similarity to the COSMIC thiopurine chemotherapy–associated mutational signature SBS87 during treatment. The T cell–inflamed GEP score for each sample is shown in Fig. 4C, and total neoepitope count, SNV mutation–derived neoepitopes, and fusion gene–derived neoepitopes were predicted for each sample and shown in Fig. 4D–F. Despite confirmed systemic drug delivery, as illustrated by the clear increase in DNA-TG, no definitive genomic or transcriptomic trends were observed between baseline and on-treatment samples.

## Discussion

TMB serves as a biomarker for predicting response to ICI therapy across multiple cancer types (22, 23), with high TMB generally associated with improved treatment outcomes. Despite its FDA approval as a tumor-agnostic biomarker, many patients with TMB-high tumors fail to respond to ICI treatment. Conversely, clinical benefit has also been observed in patients with TMB-low tumors, highlighting the limitations of TMB as a standalone biomarker and underscoring the need for further refinement and harmonization of predictive biomarker strategies (24).

The TEMPLE trial represents the first clinical investigation aiming to enhance ICI efficacy in patients with intermediate TMB (5–10 mut/Mb) through the addition of the thiopurines 6MP and 6TG.

The underlying hypothesis was that the incorporation of thioguanine nucleotides into the tumor genome would induce DNA single-nucleotide mismatching and increase mutational events, thereby increasing immunogenicity and potentially improving sensitivity to ICI therapy. This hypothesis has, in parallel, been explored in preclinical animal studies (25). Importantly, all investigational drugs were already approved and well characterized (4, 7). The thiopurine combination of 6TG and 6MP had previously been tolerated in the TEAM study (9) in combination with methotrexate, which was omitted in the present study. Furthermore, substantial overlapping toxicities between 6TG/6MP and atezolizumab were not anticipated, given their distinct mechanisms of action and established safety profiles. This formed the basis for the initial dosing strategy, in which full-dose thiopurines were combined with atezolizumab, with dose de-escalation planned only if DLTs occurred. In the phase Ib part of the study, the initial dose level proved too toxic, as all three enrolled patients developed neutropenia, including one case of febrile neutropenia and sepsis. This toxicity profile had not been observed in the TEAM study, which included 30 patients with non–high-risk ALL aged 1 to 45 years. However, this population differed substantially from the TEMPLE study population, which consisted of heavily pretreated adult patients with metastatic solid tumors and a median age of 62.5 years.

Following dose de-escalation, the subsequent dose level (DL−1: 6MP 37.5 mg/m^2^ every day + 6TG 10 mg/m^2^ every day + atezolizumab 1,200 mg every 3 weeks) demonstrated a manageable safety profile in a heavily pretreated and heterogeneous population of patients with metastatic solid tumors. At this dose level, the pharmacodynamic marker DNA-TG consistently exceeded the predefined target of 500 fmol/μg DNA, which was considered therapeutically relevant. Toxicity was tolerable, with anorexia, nausea, and fatigue being the most common treatment-related AEs. No unexpected AEs were observed. One case of SOS was suspected but not confirmed. Importantly, no SAEs related to atezolizumab, nor any organ-specific immune-related AEs (including thyroiditis, colitis, or hepatitis), were observed during the study. This was of particular interest because one concern prior to study initiation was the potential increased risk of autoimmunity through immune recognition of self-antigens and subsequent host tissue attack, as thiopurine therapy could potentially induce neoepitopes not only in cancer cells but also in normal cells.

The efficacy of the TEMPLE combination treatment with 6TG, 6MP, and atezolizumab was limited. Three patients (23%) achieved stable disease, whereas the remaining 10 patients were classified as having progressive disease. No objective responses were observed among the first 13 evaluable patients, and consequently, stage 2 of the phase II trial was not initiated, in accordance with the predefined Simon two-stage study design.

Interestingly, two patients with dendritic cell sarcoma and colorectal cancer demonstrated signs of antitumor activity, with regression of some lesions despite progression of others. However, due to overall disease progression and clinical deterioration, treatment was discontinued, and biopsies from the affected lesions could not be obtained. Considering the mechanism by which thiopurines induce random mutations, it was expected that only tumor cells acquiring mutations capable of generating immunogenic neoepitopes would respond to ICI treatment. This may explain the mixed treatment responses observed when such mutations are induced in only a subset of tumor cells and their subclones. Although these mixed outcomes did not meet the formal criteria for clinical benefit, they may indicate biologically relevant activity that warrants further investigation, suggesting that administration at earlier disease stages—when a larger proportion of tumor cells remain clonal and are therefore more likely to be uniformly affected—may be advantageous.

Looking at the genomic data, few patients were found to have tumors harboring an intermediate TMB, which made patient inclusion challenging. Most tumors with intermediate TMB were observed in breast and colorectal cancers but were otherwise distributed across a broad range of different tumor types. Patients were enrolled based on local TMB testing; however, central review using WGS and harmonized analytic methods showed that two patients were actually TMB-high (>10 mut/Mb), whereas four were TMB-low (<5 mut/Mb; Table 1; Fig. 4A). This aligns with recent studies demonstrating that the utility of TMB as a predictive biomarker is challenged by the lack of standardized assessment methodologies (4, 26). At the time of study initiation, the impact of methodologic variability on TMB assessment was not fully recognized. In retrospect, this represents an important limitation related to one of the main inclusion criteria. Nevertheless, the nationwide collaborative setup enabling inclusion based on local TMB testing was initially considered a strength, as it supported patient inclusion despite low enrollment rates. Future studies would benefit from harmonized TMB assessment within a standardized national framework.

Serial baseline and on-treatment biopsies were used to assess treatment-induced changes in the tumor mutational landscape. The biopsies were evaluated with respect to TMB and the thiopurine treatment–associated COSMIC signature SBS87. The T cell–inflamed GEP score was used to assess whether the combination treatment induced a more T cell–activated tumor microenvironment. Acknowledging that not all mutations are translated into clinically relevant neoantigens, the prediction of total neoepitope count, SNV mutation–derived neoepitopes, and fusion gene–derived neoepitopes was evaluated. However, these translational analyses were limited by the small sample size, with only seven paired samples available, and no trends or results were observed. Furthermore, thiopurines induce random single-nucleotide mutations, resulting in highly dispersed and subclonal mutations that are likely present at VAFs too low to be detected using current bioinformatic filtering strategies, which typically require a VAF of ≥5%. This limitation likely masked thiopurine-induced neoepitope generation. This could be addressed using single-cell sequencing technologies in future studies of similar design.

Several factors may explain the limited efficacy observed in the study. First, the heavily pretreated patient population with advanced disease may have had a reduced capacity to mount effective antitumor immune responses due to immune exhaustion and an immunosuppressive TME (22, 27). Second, the advanced and aggressive disease stages may have led to rapid disease progression before potential therapeutic effects of thiopurine treatment could emerge. Third, the small and highly heterogeneous cohort may have diluted potential treatment effects within responsive subgroups. A longer thiopurine priming period before the initiation of immune checkpoint inhibition could potentially have been relevant. However, this was not considered feasible in this study population with advanced and aggressive disease and limited life expectancy.

Although the TEMPLE trial represents a novel approach, several recent studies have explored other pharmacologic strategies to convert immunologically “cold” tumors into “hot” tumors. The ongoing ARETHUSA trial investigated whether temozolomide priming in microsatellite-stable (MSS) colorectal cancer could enhance sensitivity to ICI therapy (28), whereas a recent study of patients with acquired high TMB following targeted therapies in MSS colorectal cancer found that treatment-induced high TMB did not translate into meaningful responses to ICI (29). Similar findings have been reported in gliomas, in which temozolomide-induced hypermutation often fails to confer clinical benefit from ICI therapy (30). Together, these studies suggest that the etiology underlying high TMB may influence immunotherapy responsiveness. This was further supported by results from a recent DRUP study, in which mutational signature analyses indicated that high TMB associated with UV damage, APOBEC activity, or mismatch repair deficiency was more likely to correlate with ICI benefit (31). Collectively, these findings highlight the biological complexity of inducing effective antitumor immune responses in traditionally ICI-resistant cancer types.

As the TEMPLE study was initiated, new preclinical data have been published that provide proof of concept for the study. In preclinical mouse models of mutation-low melanoma, 6TG exposure induced DNA-TG integration, resulting in an increased TMB (31% increase, from 4.06 to 5.35 mut/Mb) without affecting tumor cell proliferation or cell death, thereby validating the mutagenesis approach (25). Treatment with 6TG also reshaped the tumor microenvironment by increasing the infiltration of T and NK cells. The resulting improvement in tumor control was strongly T cell–dependent and primarily mediated by CD8^+^ T cells. This enhanced immunogenicity rendered tumors more responsive to immune checkpoint blockade (anti–PD-1), significantly delaying tumor growth when combination therapy was administered.

Newly published results in immunocompetent mouse models of low-TMB melanoma demonstrated that combining thiopurines with ICI therapy promoted the formation of long-lived immune memory cell populations capable of preventing tumor regrowth (32). Notably, combined ICI therapy (anti–CTLA-4 and anti–PD-1) induced complete tumor regression in 6TG-treated mice when administered at early tumor stages, whereas efficacy declined in animals with tumors measuring 20 to 120 mm^3^ and disappeared in tumors ≥120 to 300 mm^3^ (32). These findings suggest that thiopurines combined with ICIs may be more effective in patients with less bulky disease and for remission maintenance than for remission induction.

The TEMPLE approach of combining atezolizumab with 6MP and 6TG in adult patients with metastatic solid tumors represented a novel strategy to improve the efficacy of ICI treatment. A safe and tolerable combination treatment regimen was established, and despite the lack of objective responses, further investigation of this treatment strategy may still be of interest. Future studies should preferably investigate these effects in biologically defined tumor subgroups and at earlier disease stages, potentially in combination with CTLA-4 and PD-L1 inhibition rather than PD-L1 blockade alone.

## Supplementary Material

Supplementary Table S1 Supplementary Table S1 shows the full inclusion and exclusion criteria for patients enrolled in the TEMPLE study.

Supplementary Figure S1 Supplementary Figure S1 shows DNA-TG levels measured in patients enrolled in the phase Ib and phase II parts of the TEMPLE study.

Supplementary Table S2 Supplementary Table S2 provides an overview of all adverse events reported in the phase Ib and phase II parts of the TEMPLE study.

Supplementary Table S3 Supplementary Table S3 shows the most common adverse events, occurring in >10% of patients in the TEMPLE study.

Supplementary Table S4 Supplementary Table S4 describes the representativeness of participants enrolled in the TEMPLE study.

## Data Availability

The data generated in this study are not publicly available due to restrictions related to patient privacy, informed consent, and compliance with the General Data Protection Regulation but are available from the corresponding author upon reasonable request and subject to appropriate institutional and ethical approvals.
