# Supplementary material for: A Phase Ib/II Study of Atezolizumab in Combination with Thiopurine Therapy in Patients with Metastatic Solid Tumors and Intermediate Tumor Mutational Burden
Source: Cancer Res Commun. 2026 Sep 16;6(9):2177–86. doi: 10.1158/2767-9764.CRC-26-0210 (PMC13578206; doi:10.1158/2767-9764.CRC-26-0210)
Supplement: Supplementary file 1 [file crc-26-0210_supplementary_figure_s1_suppsf1.docx]

**Supplementary Figure S1**

DNA-TG levels measured in the 3 patients treated in phase 1b (A) and in the 15 patients treated in phase 2. (B)

Thick solid line = DL0: 6MP 50.0 mg/m² QD, 6TG 12.5 mg/m² QD, atezolizumab 1200 mg Q3W

Thin solid line = RP2D/DL-1: 6MP 37.5 mg/m² QD, 6TG 10 mg/m² QD, atezolizumab 1200 mg Q3W

Dotted line = Treatment pause
