# Supplementary material for: A Phase Ib/II Study of Atezolizumab in Combination with Thiopurine Therapy in Patients with Metastatic Solid Tumors and Intermediate Tumor Mutational Burden
Source: Cancer Res Commun. 2026 Sep 16;6(9):2177–86. doi: 10.1158/2767-9764.CRC-26-0210 (PMC13578206; doi:10.1158/2767-9764.CRC-26-0210)
Supplement: Supplementary file 2 [file crc-26-0210_supplementary_table_s1_suppst1.docx]

| **Supplementary Table S1 – Inclusion and Exclusion Criteria** | |
| --- | --- |
| Inclusion Criteria | |
| The patient MUST MEET ALL the following criteria to be enrolled in the study: | |
| 1. | Signed written informed consent. |
| 2 | Age ≥ 18 years. |
| 3. | Performance status (WHO) of 0-1. |
| 4. | Histologically confirmed advanced and/or metastatic solid tumors for which standard curative measures do not exist. |
| 5. | Radiologically measurable disease according to RECIST v1.1. |
| 6. | Life expectancy estimated by the Investigator to be ≥12 weeks |
| 7. | Metastatic Lesion(s) or primary tumour accessible for biopsy. |
| 8. | Intermediate Tumor mutational burden of 5-10 mutations/mb. |
| 9. | Adequate organ function assessed by screening laboratory values:   a. Absolute lymphocyte count ≥ 0.5 x 10^9^/L b. Neutrophils ≥ 1.5 x 10^9^/L c. Platelets ≥ 100 x 10^9^/L. For patients with primary hepatocellular carcinoma platelet counts ≥65 x 10^9^/L is allowed. d. Hemoglobin ≥ 90 g/L (5.6 mmol/L) and at least 4 weeks since blood transfusion. e. Serum creatinine ≤ 1.5 × upper limit of normal (ULN) or calculated by Cockroft-Gault formula or directly measured creatinine clearance ≥ 50 mL/min at screening. f. AST ≤ 3 x ULN without, and ≤ 5 x ULN with hepatic metastasis. g. Bilirubin ≤ 1.5 x ULN (except patients with Gilbert’s syndrome, who must have total bilirubin < 3.0 mg/dL). |
| 10. | Ability to take oral medications. |
| 11. | Woman of childbearing potential must have been tested negative in a serum pregnancy test within 5 days prior to study drug initiation. |
| 12. | 1. Male and female patients who have the potential to reproduce must agree to use a highly effective method of birth control. (i.e., pregnancy rate of less than 1 % per year) during the study and for 5 months after the discontinuation of study medication. Women must refrain from donating eggs and men must refrain from donating sperm during this same period. |
|  |  |
| Exclusion criteria | |
| Any of the following: | |
| 1. | Pregnancy, lactation, or breastfeeding. |
| 2. | History or clinical evidence of central nervous system (CNS) primary tumors or metastases including leptomeningeal metastases, unless they have been previously treated, are asymptomatic, and have had no requirement for steroids or anticonvulsants in the last 14 days prior to Screening. |
| 3. | Deficiency in thiopurine methyltransferase (TPMT) or nudix hydrolase 15 (NUDT15). |
| 4. | Use, or have used, any concomitant anti-cancer medications within the previous 30 days or 5 half-lives of the medication (whichever is shortest) prior to first dose (bisphosphonates, denosumab and androgen deprivation therapies such as LHRH (GnRH) agonists are allowed if patient is on stable treatment for at least 4 weeks prior to first dose) Limited field radiotherapy for palliative purpose is allowed at any time. |
| 5. | Participants with immune-related adverse events attributed to prior immunomodulatory therapy must have resolved to Grade ≤ 1 (according to NCI CTCAE v 5.0) or baseline other than adverse events that are clinically non-significant and/or stable on supportive therapy, and are not expected to interfere with treatment in the study such as:   a. Grade ≤ 2 alopecia, asthenia, dermatologic events. b. Grade ≤ 2 anemia if hemoglobin ≥ 90 g/L (5.6 mmol/L) c. Grade 2 (> 1.5-2.0 x ULN) asymptomatic amylase and/or lipase elevation with no abdominal pain and no characteristic CT findings. However, weekly monitoring of amylase and lipase is required in this case. |
| 6. | Be an organ transplant recipient. |
| 7. | Have a history of prior other malignancy (with the exception of localized prostate cancer, adequately treated basal skin cancer or carcinoma in-situ of the cervix) within 2 years prior to first dose. |
| 8. | Have a severe autoimmune disorder requiring treatment during the last 12 months prior to first dose. Diabetes on stable anti-diabetic medication, hypothyroidism and adrenocortical deficiency on stable substitution therapy are allowed. |
| 9. | Be on chronic therapy with systemic immunosuppressant medication (inhaled, intra articular and low dose systemic corticosteroids, e.g. 7.5 or less prednisolone per day is allowed, provided that treatment has been unchanged for at least 4 weeks prior to first dose of IMP). |
| 10. | Known HIV, active hepatitis B or hepatitis C infection. |
| 11. | Participants with a history of severe allergic anaphylactic reactions to chimeric or humanized antibodies or known hypersensitivity to Chinese hamster ovary cell products or to any component of the atezolizumab formulation. |
| 12. | Any condition that, in the opinion of the Investigator, would place the patient at increased risk or preclude the patient’s compliance with the study. |
