# Supplementary material for: A Phase Ib/II Study of Atezolizumab in Combination with Thiopurine Therapy in Patients with Metastatic Solid Tumors and Intermediate Tumor Mutational Burden
Source: Cancer Res Commun. 2026 Sep 16;6(9):2177–86. doi: 10.1158/2767-9764.CRC-26-0210 (PMC13578206; doi:10.1158/2767-9764.CRC-26-0210)
Supplement: Supplementary file 3 [file crc-26-0210_supplementary_table_s2_suppsf2.docx]

| **Supplementary Table S2 – Overview of Adverse Events** | | | | |
| --- | --- | --- | --- | --- |
|  |  | DL0  (n=3) | DL-1 (RP2D)  (n=15) | All patients  (n = 18) |
| Adverse events (including SAE, n (%) | All | 22 | 96 | 118 |
|  | Grade 1 and 2 | 19 (86.4) | 88 (91.7) | 107 (90.7) |
|  | Grade =>3 | 3 (13.6) | 8 (8.3) | 11 (9.3) |
| Adverse events related to study treatment: 6TG and 6MP, n (%) | Related | 8 (36.4) | 18 (18.8) | 26 (22.0) |
|  | Not related | 14 (63.6) | 78 (81.2) | 92 (78.0) |
| Adverse events related to study treatment: atezolizumab, n (%) | Related | 2 (9.1) | 4 (4.2) | 6 (5.1) |
|  | Not related | 20 (90.9) | 92 (95.8) | 112 (94.9) |
| Action taken of adverse events, n (%) | No Change | 20 (90.9) | 90 (93.9) | 110 (93.2) |
|  | Drug dose changed | 1 (4.5) | 0 (0.0) | 1 (0.8) |
|  | Drug interruption | 0 (0.0) | 2 (2.1) | 2 (1.7) |
|  | Drug permanently discontinued | 1 (4.5) | 4 (4.2) | 5 (4.2) |
| SAE, n (%) |  | 2 | 7 | 9 |
| SAE related to study treatment: 6TG and 6MP, n (%) | Related | 2 (100.0)* | 1 (14.3)* | 3 (33.3) |
|  | Not related | 0 (0.0) | 6 (85.7) | 6 (66.7) |
| SAE related to study treatment: atezolizumab, n (%) | Related | 0 (0.0) | 0 (0.0) | 0 (0.0) |
|  | Not related | 2 (100.0) | 7 (100.0) | 9 (100.0) |
| Adverse events were defined as treatment-emergent adverse events occurring after treatment initiation (C1D-7), or as a worsening of pre-existing adverse events.  *SAE related to 6TG and 6MP in dose level D0 were DLTs febrile neutropenia and sepsis. SAE related to 6TG and 6MP in dose level D1/RP2D was a patient with suspected SOS (hepatic sinusoidal obstruction syndrome).  Abbreviations: DL0: dose level 0 (6MP 50 mg/m² once daily + 6TG 12.5 mg/m² once daily + atezolizumab 1200 mg every 3 weeks); DL–1: dose level –1 (6MP 37.5 mg/m² once daily + 6TG 10.0 mg/m² once daily + atezolizumab 1200 mg every 3 weeks); RP2D: recommended phase 2 dose; SAE: serious adverse event; 6TG: 6-thioguanine; 6MP: 6-mercaptopurine. | | | | |
