# Supplementary material for: A Phase Ib/II Study of Atezolizumab in Combination with Thiopurine Therapy in Patients with Metastatic Solid Tumors and Intermediate Tumor Mutational Burden
Source: Cancer Res Commun. 2026 Sep 16;6(9):2177–86. doi: 10.1158/2767-9764.CRC-26-0210 (PMC13578206; doi:10.1158/2767-9764.CRC-26-0210)
Supplement: Supplementary file 4 [file crc-26-0210_supplementary_table_s3_suppsf3.docx]

| **Supplementary Table S3 - Most common Adverse Events (>10% in total patients)** | | |
| --- | --- | --- |
| Term | All Grades | Grade ≥ 3 |
| Pain, n (%) | 12 (67 %) |  |
| Fatigue, n (%) | 9 (50 %) | 1 (6 %) |
| Anorexia, n (%) | 8 (44 %) |  |
| Nausea, n (%) | 8 (44 %) |  |
| Constipation, n (%) | 6 (33 %) |  |
| Headache, n (%) | 5 (28 %) | 1 (6 %) |
| Vomiting, n (%) | 5 (28 %) |  |
| Cough, n (%) | 4 (22 %) |  |
| Dry mouth, n (%) | 4 (22 %) |  |
| Rash, n (%) | 4 (22 %) |  |
| Diarrhea, n (%) | 3 (17 %) |  |
| Edema, n (%) | 3 (17 %) |  |
| Stomatitis, n (%) | 3 (17 %) |  |
| Ascites drainage, n (%) | 2 (11 %) | 2 (11 %) |
| Dizzyness, n (%) | 2 (11 %) |  |
| Fever, n (%) | 2 (11 %) |  |
| Dysguesia, n (%) | 2 (11 %) |  |
| Dyspepsia, n (%) | 2 (11 %) |  |
| Dyspnea, n (%) | 2 (11 %) |  |
| Flushing, n (%) | 2 (11 %) |  |
| Pruritus, n (%) | 2 (11 %) |  |
| Urinary tract infection, n (%) | 2 (11 %) |  |
| Weightloss, n (%) | 2 (11 %) |  |
| Biochemistry*: |  |  |
| Liver function tests**, n (%) | 4 (22 %) | 3 (17 %) |
| Neutrophil count decreased, n (%) | 3 (17 %) ** | 1 (6 %)** |
| Anemia, n (%) | 8 (44 %) |  |
| *Data from platelet count and bilirubin were also collected but were not defined as common adverse events (>10% in total patients). **Liver function tests cover all reported CTCAE-defined liver toxicities, including transaminase increases, sinusoidal obstruction syndrome, and GGT elevation. ***All incidents of neutropenia were observed in the phase 1 patients receiving the high starting dose D0 | | |
