# Supplementary material for: A Phase Ib/II Study of Atezolizumab in Combination with Thiopurine Therapy in Patients with Metastatic Solid Tumors and Intermediate Tumor Mutational Burden
Source: Cancer Res Commun. 2026 Sep 16;6(9):2177–86. doi: 10.1158/2767-9764.CRC-26-0210 (PMC13578206; doi:10.1158/2767-9764.CRC-26-0210)
Supplement: Supplementary file 5 [file crc-26-0210_supplementary_table_s4_suppsf4.docx]

| **Supplementary Table S4 - Representativeness of Study Participants** | |
| --- | --- |
| Cancer type(s)/subtype(s)/stage(s)/  condition | Adult patients with advanced solid tumors without remaining standard treatment options were included. The cohort was heterogeneous, with breast and colorectal cancer most common, reflecting population incidence. Rare cancers (e.g., dendritic cell sarcoma and large cell neuroendocrine lung carcinoma) were also included and are typically underrepresented in clinical studies. Intermediate TMB occurs across multiple tumor types, representing a group of patients who often have limited response to immune checkpoint inhibitors (3). |
| Considerations related to: |  |
| Sex | Females comprised 10 (55.6%), yielding a near-balanced distribution. This slight predominance of women in the study is expected, as breast cancer—one of the most common diagnoses in the cohort—occurs almost exclusively in females. , |
| Age | Median age was 62.5 years, lower than the general cancer population, reflecting strict inclusion criteria and phase 1 selection bias. |
| Race/ethnicity | All participants were Caucasian, representative of the Danish population. Generalizability to more diverse populations is uncertain due to limited data on TMB distribution across ethnic groups independent of cancer type. |
| Geography | This was a nationwide Danish study. Although conducted within a small geographic area, it ensures a homogeneous population with equal access to healthcare and expensive cancer treatments through a tax-funded system, reducing disparities and supporting representativeness. |
| Other considerations | A small exploratory phase 1/2 study evaluated safety, adverse events, and dosing in a heavily pretreated metastatic population. Strict inclusion criteria (TMB 5–10 mut/Mb, good performance status, adequate organ function) limited recruitment. A more homogeneous cohort was not feasible due to the rarity of eligible patients and the exploratory design. |
| Overall representativeness of this study | The study population represents a highly selected group of cancer patients, comprising heavily pretreated Danish patients with advanced solid tumors referred for experimental treatment. They are younger and fitter than the general cancer population at a similar disease stage. The heterogeneity of tumor types reflects that intermediate TMB occurs across multiple cancers, supporting a tumor-agnostic approach, though limiting conclusions within individual cancer types while enabling inclusion and investigation of rare cancers. |
